# A Novel Method for Training Mice in Visuo-Tactile 3-D Object Discrimination and Recognition

**DOI:** 10.3389/fnbeh.2018.00274

**Published:** 2018-11-13

**Authors:** Xian Hu, Ogaga Urhie, Kevin Chang, Rachel Hostetler, Ariel Agmon

**Affiliations:** Department of Neuroscience, West Virginia University School of Medicine, Morgantown, WV, United States

**Keywords:** object discrimination, object recognition, object memory, operant conditioning, food restriction, whisker trimming, mice

## Abstract

Perceiving, recognizing and remembering 3-dimensional (3-D) objects encountered in the environment has a very high survival value; unsurprisingly, this ability is shared among many animal species, including humans. The psychological, psychophysical and neural basis for object perception, discrimination, recognition and memory has been extensively studied in humans, monkeys, pigeons and rodents, but is still far from understood. Nearly all 3-D object recognition studies in the rodent used the “novel object recognition” paradigm, which relies on innate rather than learned behavior; however, this procedure has several important limitations. Recently, investigators have begun to recognize the power of behavioral tasks learned through reinforcement training (operant conditioning) to reveal the sensorimotor and cognitive abilities of mice and to elucidate their underlying neural mechanisms. Here, we describe a novel method for training and testing mice in visual and tactile object discrimination, recognition and memory, and use it to begin to examine the underlying sensory basis for these cognitive capacities. A custom-designed Y maze was used to train mice to associate one of two 3-D objects with a food reward. Out of nine mice trained in two cohorts, seven reached performance criterion in about 20–35 daily sessions of 20 trials each. The learned association was retained, or rapidly re-acquired, after a 6 weeks hiatus in training. When tested under low light conditions, individual animals differed in the degree to which they used tactile or visual cues to identify the objects. Switching to total darkness resulted only in a transient dip in performance, as did subsequent trimming of all large whiskers (macrovibrissae). Additional removal of the small whiskers (microvibrissae) did not degrade performance, but transiently increased the time spent inspecting the object. This novel method can be combined in future studies with the large arsenal of genetic tools available in the mouse, to elucidate the neural basis of object perception, recognition and memory.

## Introduction

The uncanny ability of humans and other primates to recognize a familiar 3-dimensional (3-D) object by vision or by touch in less than two tenths of a second (Thorpe et al., 1996; Hung et al., 2005; Liu et al., 2009; Gurtubay-Antolin et al., 2015), regardless of lighting, viewing angle, distance or other changing conditions, has long fascinated philosophers, psychologists, computer scientists, and both experimental and computational neuroscientists (Bulthoff and Edelman, 1992; Logothetis and Sheinberg, 1996; Tarr and Bulthoff, 1998; Riesenhuber and Poggio, 2002; Palmeri and Gauthier, 2004; Serre et al., 2007; Hoffman and Logothetis, 2009; Ungerleider and Bell, 2011; DiCarlo et al., 2012). Several different experimental procedures have been developed to examine the neural basis of object recognition, discrimination and memory. A common object recognition paradigm, used in primate studies for over half a century, is the “Delayed Match/Non-Match to Sample” (DMS/DNMS) test (Mishkin et al., 1962; Mishkin and Delacour, 1975; Zola-Morgan et al., 1989). In this procedure an object is first presented to the animal for sampling and, after a variable delay, presented again together with a different object. The animal is rewarded for selecting the matching object (in DMS experiments) or the non-matching object (in DNMS experiments), thus presumably demonstrating “recognition,” i.e., association of the object with a previously stored memory of the same object. A few early studies adapted the DNMS paradigm, or variants thereof, to rats (Aggleton et al., 1986; Rothblat and Hayes, 1987; Mumby et al., 1990; Jackson-Smith et al., 1993). However, the great majority of 3-D object recognition studies in the rat, and to our knowledge all in the mouse, have used the novel object recognition (NOR) test, also called spontaneous object recognition (SOR) or one-trial object recognition (OTR) test (Ennaceur and Delacour, 1988; Ennaceur et al., 1989; Ennaceur and Meliani, 1992). While superficially similar to the DNMS paradigm, in the NOR test the animal is not trained; rather, the experiment relies on the natural tendency of rodents (and other species) to spend more time exploring a novel object in preference to a previously encountered one (reviewed in Dere et al., 2007; Winters et al., 2008; Antunes and Biala, 2012; Blaser and Heyser, 2015).

While the NOR procedure has been widely used for studying the neuropsychology of object recognition memory—i.e., its neuroanatomical and pharmacological basis (Brown et al., 2012; Warburton and Brown, 2015)—it has recently been criticized for its low reproducibility between labs (Akkerman et al., 2012; Cohen and Stackman, 2015) and for being prone to a variety of misinterpretations (Gaskin et al., 2010; Gervais et al., 2013). Also, the standard NOR test does not work with retention delays of more than a few hours, although a small number of studies used a modified version of the NOR test to demonstrate retention for several weeks (Mumby et al., 2002; Broadbent et al., 2010), and a recently developed modified DNMS task demonstrated retention of object recognition for nearly a year (Cole et al., 2018). Importantly, neither the NOR nor the DNMS task are suitable for repeated testing of the same animal on the same set of objects as would be needed, for example, for elucidating the sensorineural mechanisms underlying object perception and discrimination, or for probing the psychophysical limits to the animal’s object discrimination abilities. Lastly, NOR cannot be used to study how neural representations of objects are formed in the course of learning to recognize an object, precisely because NOR is not based on learning. All of the above questions are best studied by training the animal in object discrimination and recognition using operant conditioning (behavioral reinforcement) methods. Indeed, much of our knowledge about the cognitive psychology of object perception and recognition comes from experiments on primates (reviewed by Op de Beeck and Baker, 2010; Hirabayashi and Miyashita, 2014), pigeons (Soto and Wasserman, 2014) and rats (Zoccolan, 2015) trained to discriminate between, or conversely to associate, specific 3-D objects or 2-D images of objects. To-date, however, no such studies have been reported in mice.

In the last two decades, the rapidly expanding repository of genetically modified mice, together with the burgeoning arsenal of genetically targetable tools for recording, imaging and manipulating neuronal activity, have made the mouse the species of choice for studying the nervous system at the cellular, circuit and systems levels (O’Connor et al., 2009; Zeng and Madisen, 2012; Petersen and Crochet, 2013). Consequently, higher cognitive functions such as perceptual decisions, goal-directed behavior, attention and working memory, previously studied mostly in primates (Goldman-Rakic, 1996; Fuster, 2000; Romo and Salinas, 2003; Shadlen and Kiani, 2013), are increasingly being studied in mouse models (Huberman and Niell, 2011; Harvey et al., 2012; Carandini and Churchland, 2013; Guo et al., 2014; Allen et al., 2017). However, studies of the psychophysical, cognitive and neurophysiological basis of 3-D object perception and recognition in mice are conspicuously absent. Our main goal in the present study was therefore to develop an easily implementable procedure for training and testing mice in 3-D object discrimination, recognition and memory, thereby opening up this field to the powerful tools available in mice. Our second goal was to begin to examine the sensory modalities employed and sensory cues utilized by mice to perceive, discriminate between and recognize 3-D objects.

## Methods

### Animals and Food Restriction

All methods were approved by the WVU Institutional Animal Care and Use Committee and followed United States Public Health Service guidelines. We trained two cohorts of mice, an initial pilot cohort of five and a main cohort of four C57BL/6J male mice (Charles River Laboratories), 6–8 week old at the beginning of training. Upon arrival, animals were housed under a reverse light cycle (lights off at 8 am, lights on at 8 pm). Animals in the main cohort were uniquely marked by performing an ear punch on one or both ears, thus allowing their rapid identification in low light as “Right,” “Left,” “Both,” or “Neither” (R, L, B, N, respectively). The mice were habituated to handling and then placed on a food restriction regime, aiming to maintain their weight at 80–85% of its baseline value, the latter determined by averaging their initial weight over three consecutive days. To achieve the target weight, each animal was fed a fixed amount of rodent chow per day (typically 3.5 g), and its weight monitored daily. Training did not begin until the animal’s weight stabilized within the desired range. Once training started, animals were weighed twice daily, before training and after training and feeding. When an animal’s weight deviated from target, the amount of food was incremented or decremented by ∼0.25 g at a time. Since mice are still growing during the first 20 weeks of life (Fu et al., 2013), we used standard C57Bl/6J age-weight charts^1^ to adjust the baseline weight (and thereby the target weight), increasing the baseline weight by 1 g per week until 12 weeks of age, and then by 0.5 g per week until 20 weeks of age.

### Objects

Mice were trained to discriminate between two canonical 3-D objects—a cube and a tetrahedron. The objects had 30 and 37 mm long edges, respectively, and were of equal height. Both shapes consist of flat surfaces joined by sharp edges, making them more challenging to discriminate. Several identical sets of the two objects were constructed on the same 3-D printer from identical material, so all had the same color, texture and presumably olfactory cues. A set of objects was placed in the home cage a week before training began, to familiarize them to the animals; the position of the objects within the cage was routinely shuffled to encourage exploration.

### Training Arena

**FIGURE 1:**
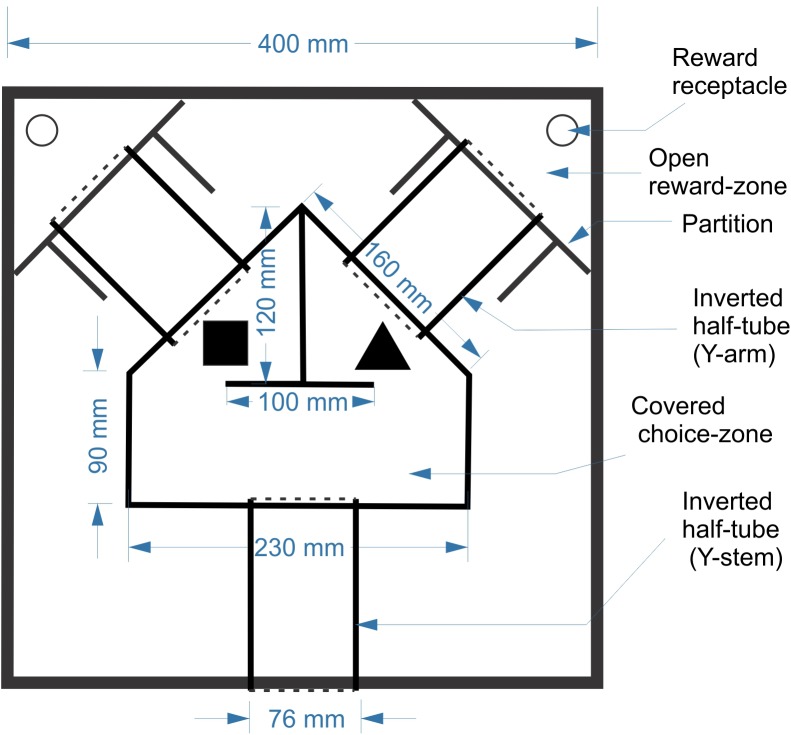
Schematic of the training arena and maze.

Animals were trained in a custom-designed maze placed inside a 40 cm (L) × 40 cm (W) × 25 cm (H) open arena made of ¼” (6.3 mm)-thick sheets of acrylic (Figure 1). The maze itself was constructed from three segments of cast acrylic tubes (3′′ (75 mm) outer diameter, 2.5′′ (63 mm) inner diameter), cut in half longitudinally and placed cut-side down on the floor of the arena, forming tunnels. The three tunnels were contiguous with a central covered pentagonal “choice zone” made of the same acrylic sheets as the arena; round openings in the acrylic sheets allowed the choice zone to fit snugly over the tunnels. The tunnels and choice zone together formed a continuous, enclosed Y-shaped maze, held down only by its own weight. The arena entry opening, at the bottom center of the lower wall, could be blocked by a guillotine door to prevent the mouse from escaping. Two additional guillotine doors could block entry to the two arms of the Y during early phases of training. Two self-supporting acrylic partitions, at the end of the two Y arms, fenced off the far corners of the arena, creating triangular “reward zones.” Two doors made of flexible transparency sheets allowed one-way passage from the Y arms into the reward zones, but not back. Thus, once the mouse entered the reward zone it was trapped in the corner, enabling its retrieval by the experimenter. The two objects were placed inside the choice zone, one in front of the entrance to each arm. The exact orientation of each object was allowed to vary randomly each time, making it unlikely that the animals could identify objects based on unique marks on their surfaces. As a reward, a small piece of Kellogg’s FrootLoop (1/16 piece, ∼16 mg) was placed in the reward zone corresponding to the assigned object. To equalize olfactory cues, both reward zones contained a large piece of FrootLoop placed inside an inverted plastic bottle cap and covered by a perforated aluminum foil, making it inaccessible to the animal. The arena was placed in a training room separated by closed doors from the main room in which the mice were housed and fed and from the control room which housed the computer.

### Behavioral Training

Behavioral training was done in four stages. In Stage 1, the mice were habituated to the arena and the maze over 3 days, with no objects or rewards present. In Stage 2 they were released into the maze through the entry door, and were rewarded for going all the way through either arm of the arena without turning back. In Stage 3 mice were trained to associate one of the two objects (the S^+^ or “assigned” object) with a reward; object assignment was balanced between the four animals. The assigned object was placed in the choice zone, in front of the entry to one of the arms; the other arm was blocked by the guillotine door, and the reward zone at the end of the open arm was baited with a reward. Finally, Stage 4 was the actual object discrimination training. In this stage both objects were placed in the choice zone, one in front of each arm, with both arms open but only the arm behind the assigned object leading to a reward. In Stages 3 and 4, the rewarded side (R or L) was alternated between trials in a predetermined, quasi-random sequence which was changed between sessions. The sequences included both sides with equal probability and with no more than three consecutive repetitions of the same side. In a small number of sessions, deviations from the predetermined sequences were employed to correct for animal bias (i.e., rewarding one side more often if the animal was consistently choosing the other side). *Post hoc* analysis of our sequences showed that a strategy of simple alternation (R-L-R-L-…) would have resulted in ≤60% of correct trials, as indicated.

Each Stage 4 trial was initiated by manually releasing the mouse into the maze through the entry door. Within the choice zone, the mouse could “change its mind” after sampling one of the objects and switch to the other side; however, once it entered a reward zone, the one-way door prevented it from going back. The animal was then retrieved and placed in a clean holding cage while the experimenter switched objects (if needed) and re-baited the arena. Mice were trained during a consistent time window of their dark cycle for 20 trials/session. Mice in the second cohort were trained for 1 session/day, 5 days/week (in the first cohort, 1–2 sessions/day and 6 days/week). The arena and objects were wiped thoroughly with 70% ethanol before and after each session. A performance score (PS) for each session was calculated as the percentage of trials in which the animal entered the correct reward zone. Once a mouse performed at ≥80% for three consecutive sessions it was considered to have reached criterion, and subsequent sessions were considered “testing.”

### Lighting and Video Recordings

All training and testing sessions were recorded with an IR digital video camera (Ikegami SC46) mounted above the arena. In most sessions the IR light source (Axton AT-8SB, 850 nm wavelength) was placed in a translucent box under the arena, rendering the animals as sharp silhouettes. Because of the need to share the arena with a different project, some of the later sessions were recorded with the IR lighting mounted above the arena, which unfortunately introduced reflections and degraded the contrast (e.g., Figure 5, bottom right). Videos were acquired and analyzed using Ethovision XT software (Noldus^2^). The program was run from a computer located in an adjacent room, with the video signal passed through cables in the ceiling.

Training and testing were initially done under light from an overhead fluorescent bulb filtered through a semi-transparent orange-red pane, allowing the experimenter to directly observe the trial. Illuminance of the arena floor under these conditions was ≤26 lux, as measured with a Gossen Mavolux 5032C illuminance meter with a wavelength response adjusted to human vision. Later sessions were performed in total darkness (other than the IR light), which required not only switching off the overhead lights but also masking any LEDs incorporated into the camera or the other electronic equipment in the room. Under these conditions, the illuminance meter registered 0 lux and the arena and the objects were not visible even to a dark-adapted human observer.

### Whisker Manipulations

To test the role of whisker-dependent cues in object discrimination, we removed the large whiskers (“macrovibrissae”) and/or the smaller anterior whiskers (“microvibrissae”) on one or both sides of the snout. Anesthesia was induced by 3% isoflurane and then maintained by 1.5% isoflurane, administered through a nose cone (fashioned from the rubber bulb of a Pasteur pipette, cut diagonally). Macrovibrissae were visualized under a stereomicroscope and cut at their base, as close as possible to the skin, using small surgical scissors. Microvibrissae were removed by a depilating cream (“Nair”), applied with a Q-tip and left for 5–6 min, then carefully removed and the skin rinsed with clean water. Care was taken not to allow the cream to contact mucous tissues, which meant that the microvibrissae inside the buccal pad were left intact. Animal was allowed to recover from anesthesia for at least 1.5 h before behavioral testing. Trimming and depilation were typically repeated every other day.

### Data Analysis

Videos were played back and analyzed in Ethovision. Animal trajectories through the maze (Figure 4) were traced by overlaying a trail of the machine-identified nose point, corrected by hand when necessary, over a tracing of the arena as it appeared in a captured video frame. Object inspection strategies (Figure 5) were based on the animal’s behavior while inspecting the wrong object (cube) during side reversal trials. Trials in which the mouse climbed on the cube with all four legs were excluded from analysis; this happened mostly with one animal (N) under red light conditions. Object inspection duration (Figure 6) was quantified by counting all video frames in which the animal’s snout pointed toward the object and was within whisker touch from it. Frames in which the animal was clearly disengaging from the object, even if it was still within touching distance, were not counted.

## Results

### A Novel Operant Conditioning Method for Training Mice in Object Discrimination and Recognition

Using operant conditioning and a custom-designed Y maze, we trained mice to discriminate between two objects, a cube and a tetrahedron. Our original intent was to force the animals to rely mostly on tactile cues, as do mice and other small nocturnal and subterranean mammals in the wild (Anjum et al., 2006; Diamond et al., 2008; Catania, 2012; Roohbakhsh et al., 2016). Consequently, initial training was done under dim orange-red light to which mice, lacking red cone opsins, are an order of magnitude less sensitive than humans (Jacobs et al., 2004; Peirson et al., 2017). The mouse encountered the two objects at the entry to the two arms of the Y, and was free to sample both objects before entering one of the two arms. A PS equal to the fraction of trials in which the correct reward zone was entered was calculated for each 20-trial session.

**FIGURE 2:**
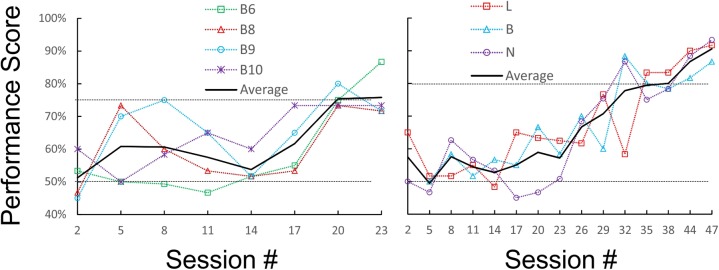
Learning curves of the pilot **(Left)** and main **(Right)** cohorts during initial training under orange-red light. Raw performance scores for each animal (averaged in blocks of three consecutive sessions) are plotted as a dotted line, and the average of all animals is plotted as a solid line. The chance (50%) and criterion (75 and 80%) levels are indicated by dotted horizontal lines.

We first tested and optimized the maze, the food restriction regime and the training method on a pilot cohort of five mice, trained for 3 weeks and 31 sessions. Four of the five mice reached our earlier performance criterion, of ≥75% correct trials for three sessions in a row, by the 19th, 21st, 22nd, and 23rd sessions (criterion sessions included), respectively (Figure 2, left panel); a fifth animal failed to reach this criterion within this training period. Based on these results, we embarked on training and testing the main cohort of four mice, which were trained, and then tested, for up to 7 months and 114 sessions. For this cohort we chose a more stringent performance criterion, of ≥80% correct trials for three sessions in a row. Three of the animals (B, N, and L) achieved this criterion in 30, 33, and 37 sessions (criterion sessions included), respectively (Figure 2, right panel). Once at criterion, performance stayed at or above 80% except for occasional “dips.” The fourth animal did not reach criterion after 50 training sessions, and was removed from the experimental cohort.

**FIGURE 3:**
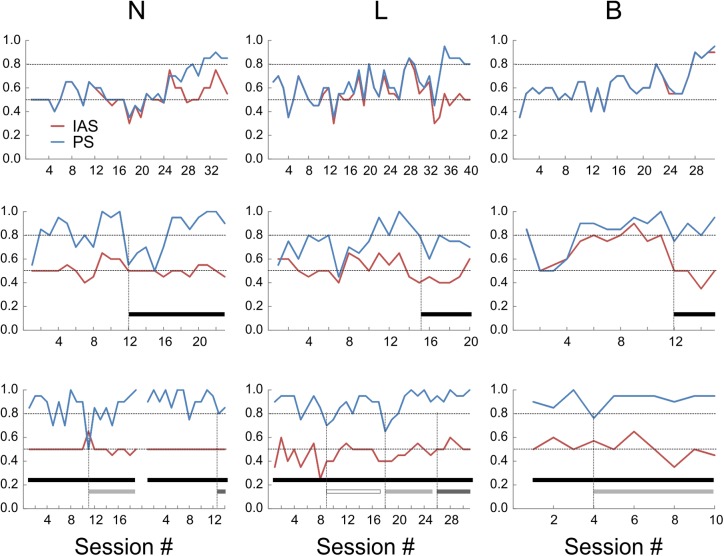
Performance scores (PS, blue lines) and initial approach scores (IAS, red lines) of the three animals under different sensory environments. Upper panels: under orange-red light. Middle panels: in total darkness (black bars). Lower panels: in total darkness, with large whiskers trimmed unilaterally (open bars) or bilaterally (light-gray bars), or with both whiskers and microvibrissae removed (dark-gray bars). The break in the lines in the lower left panel represents a 3-week hiatus in testing during which whiskers were allowed to regrow; session numbering restarts after the break.

It was possible that the mice were choosing sides based on inadvertent cues generated by the experimenter rather than on object identity. Moreover, with some stimulus sequences, it may be possible for subjects in a two-alternative discrimination experiment to “cheat” and achieve above-chance performance by following a predetermined order or an outcome-based strategy, without actually discriminating between the stimuli (Gellerman, 1933; Levine, 1963; Fellows, 1967). To determine if mice were actually sampling the objects before making a choice, we examined the videos of the test sessions (the sessions after reaching criterion). In videos from two of the mice (N and L), in about half of the “hit” trials the animals directly approached the correct object and immediately proceeded to collect the reward, but in the remaining trials the animal initially approached the wrong object, explored it momentarily and then switched to the correct object. Such “side reversals” implied that the animals were indeed making decisions based on the perceived object identity, and using short-range cues (most likely tactile, but possibly also visual) to determine this identity. In contrast, mouse B almost never exhibited side reversals, instead going directly to the correct side, suggesting that this animal was using long-range cues (most likely visual) to discriminate between the two objects. To quantify these contrasting behaviors we calculated an “Initial Approach Score” (IAS) for each session, equal to the fraction of trials in which the first object approached by the animal was the correct one. Figure 3 (top panels) illustrates the evolution of the PS and IAS (blue and red lines, respectively) for each animal during the initial training period under orange-red lights (these are the same sessions illustrated in Figure 2). As evident from these plots, in mice N and L the two curves diverged once the animal learned to discriminate the two objects, with the PS gradually increasing to criterion and the IAS remaining at or slightly above the chance level. In the third animal (B) the IAS improved in-step with the PS and the two curves were totally overlapping, suggesting that this mouse made a decision before entering the choice zone and without sampling the objects at close range.

Previous studies found that rats trained in visual object discrimination retain object memories when retested up to 12 weeks later (Mumby et al., 1999). To test for retention of remote object memory, all three mice were given a 6 weeks hiatus in the testing and food restriction regime. Food restriction was then re-instated and, once animal weights stabilized, testing was resumed. Performance recovered to criterion within 1 (N), 4 (B), and 11 (L) sessions under red-orange light (Figure 3, middle panels; session #1 is the first session after testing was resumed). This indicates robust retention of object memory in one animal, and at least partial retention in the other two mice, allowing them to rapidly re-acquire the discrimination performance. As before, the IAS of B closely tracked its PS, while IAS stayed at chance level for N and L.

### Sensory Cues Used for Object Discrimination and Recognition

The finding that mouse B was able to discriminate between the objects at far range was unexpected, given that the experiments were done under dim orange-red light to which mice are relatively insensitive (Peirson et al., 2017). To test whether the mice were using visual or tactile cues, we retested their performance in total darkness (Figure 3, middle panels; black bars indicate sessions in total darkness). In total darkness (i.e., under IR light only), PS for N transiently dropped to chance level, but recovered to criterion within six sessions of continued testing. The performance of L in total darkness degraded to below criterion for about 20 sessions (only the first six sessions illustrated in Figure 3) before fully recovering to criterion. This suggested that these two animals were initially relying, at least to some extent, on close-range visual cues, but rapidly adapted to total darkness, presumably switching to purely tactile cues. The performance of B in total darkness remained at criterion with nearly no degradation, however, its IAS fell to chance level. Thus, under total darkness this animal presumably switched its discrimination strategy instantaneously, from relying on long-range visual cues to relying on short-range tactile cues.

Tactile sensation in mice can potentially be mediated by several distinct sensory organs, including glabrous skin (e.g., paws), large mystacial whiskers (macrovibrissae) and the more anterior, shorter microvibrissae (Brecht et al., 1997; Kuruppath et al., 2014). To test for use of macrovibrissae, we retested the three mice in total darkness after trimming the large whiskers under light anesthesia (Figure 3, lower panels). In N and B, macrovibrissae were trimmed bilaterally (light gray bars). Performance of N transiently dipped to chance level for one session, followed by recovery over five sessions to ≥90%. Performance of B dipped on the 1st day slightly below criterion but immediately recovered and remained ≥90%. In L, whiskers were initially trimmed unilaterally (open bar), causing a transient two-session dip below criterion, followed by performance at criterion for the next seven sessions. Subsequent bilateral trimming of all whiskers (light gray bar) had a similar effect—a transient two-session dip below criterion, followed by consistent performance at ≥90% for the next five sessions. Since trimming was repeated at least every other day, the initial dip in performance could not be attributable to lingering anesthesia effects. Lastly, in two of the animals we combined macrovibrissae trimming with depilation (using “Nair”) of most of the microvibrissae (Figure 3, lower panels, dark gray bars). In N, the previously trimmed large whiskers were allowed to re-grow for 3 weeks and the animal was then retested for 12 sessions. Both the large whiskers and the microvibrissae were then removed and the animal retested for two sessions. Performance stayed at ≥80%. In L, microvibrissae were removed immediately following 12 sessions of bilateral macrovibrissae trimming and the animal retested for two sessions; performance remained at ≥90%.

**FIGURE 4:**
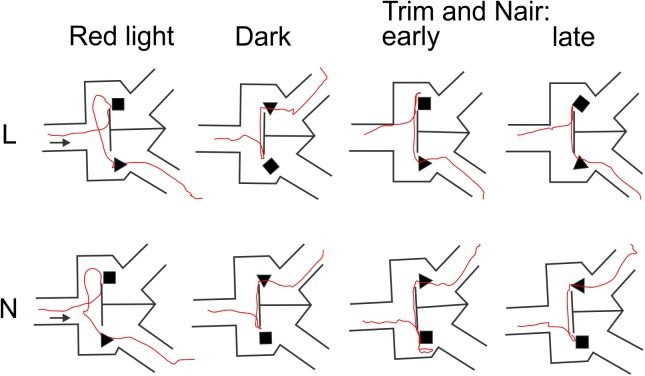
Representative animal trajectories through the maze under different conditions. The assigned object for both mice was the tetrahedron. The “early” and “late” trajectories correspond to earlier and later trials after trimming the macrovibrissae and depilating the microvibrissae.

**FIGURE 5:**
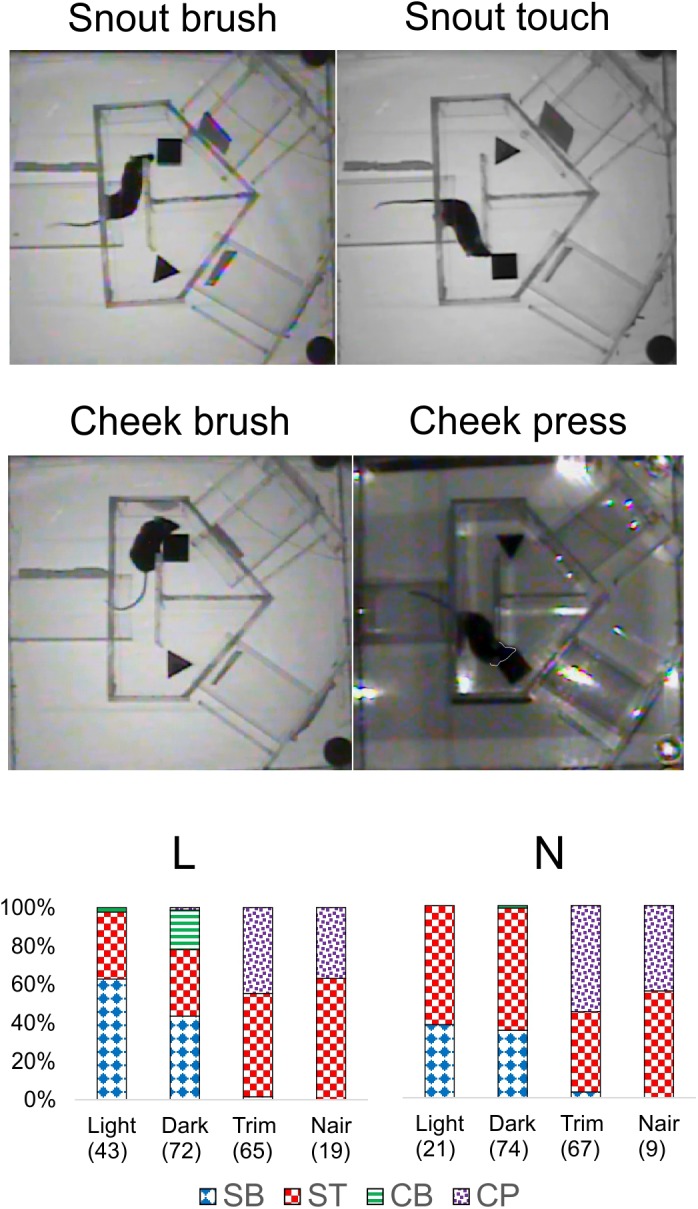
Object inspection strategies used by the mice. *Upper and middle panels*, single video frames depicting the animal’s closest approach to the object during side reversal trials representative of each strategy. The animal’s snout in “Cheek press” is outlined by a white line as a visual aid. *Lower panels*, the fraction of trials in which each mouse used each strategy, pooled from 8 to 10 consecutive sessions under each sensory condition and the two Nair sessions. Number of trials indicated in parenthesis. Trials in which the mouse climbed on the cube (mostly N in the red light sessions) were excluded. SB, snout brush; ST, snout touch; CB, cheek brush; CP, cheek press.

**FIGURE 6:**
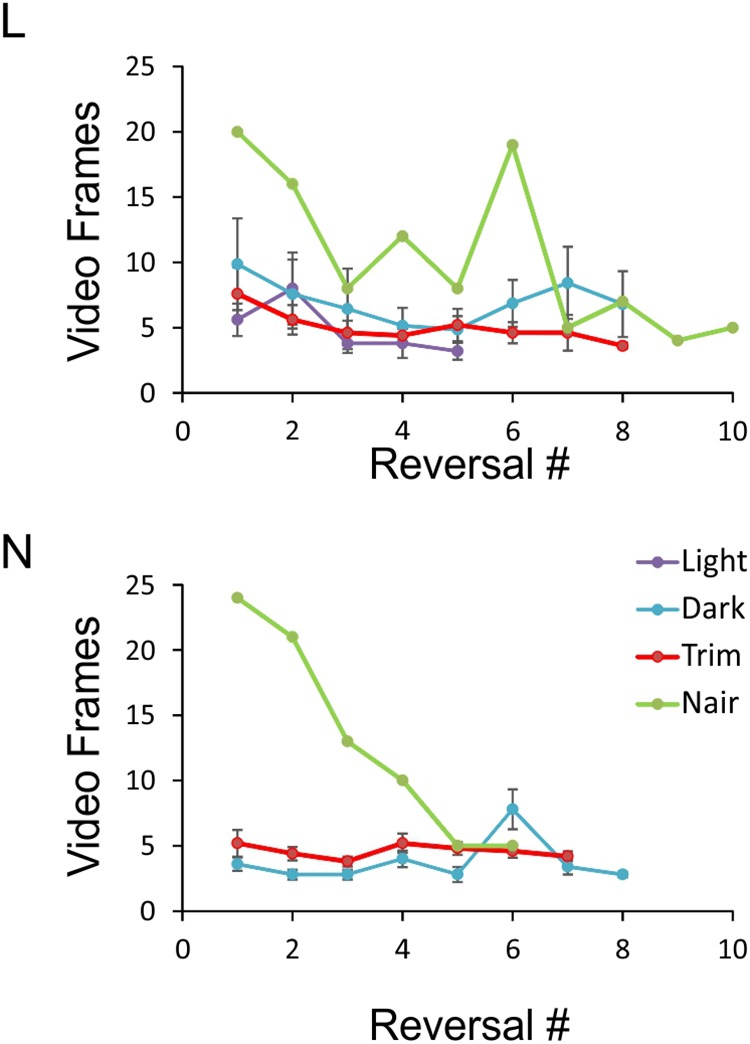
Object exploration time under the four sensory conditions. Data points represent the number of video frames in each side reversal trial during which the animal was inspecting the object. Trials in which the animal climbed on the object were excluded, and the remaining trials ordered chronologically. For each condition, at least five sessions with at least five side reversals each are averaged, except for the Nair data points which represent a single session. Note that because of N’s tendency to climb on the object, there were not enough sessions in red light to analyze. Error bars represent SEM.

That whisker and microvibrissae removal had little or no effect on performance does not necessarily mean that these sensory organs (when present) were not being used by the mice for object discrimination. Rather, after their removal the animals could have rapidly adopted alternative sensory exploration strategies. To examine such alternative strategies, we analyzed video recordings of representative trials from the four different sensory conditions (red light, total darkness, trimmed whiskers and “Naired” microvibrissae). We paid special attention to side reversal trials, since they offered an opportunity to observe the animal as it inspected the wrong (S^-^) object before reversing course. Trials in which the mouse went directly to the assigned (S^+^) object were much less informative, because in these cases the animal brushed by the object and proceeded without any perceptible pause to the reward zone. Figures 4–6 focus on the two mice (L and N) which had the tetrahedron as the S^+^ object, and therefore inspected the cube during side reversal trials. Plotting the trajectory of the animal’s nose point during representative reversal trials (Figure 4), and examining individual video frames of the animal’s posture at its closest approach to the object (Figure 5), revealed changes in exploratory strategies under different sensory conditions. With intact whiskers in either red light or total darkness, mice made their choice during a momentary close approach to the object, sometimes as short as one video frame (≤33 ms), either touching their snout to the object or without a snout touch but with the whiskers presumably making contact with the object. We call these strategies “snout touch” and “snout brush,” respectively (Figure 5, upper panels; note that the whiskers were below the resolution of our analog videos and are therefore not visible). The lack of actual snout contact during snout brush is also seen from the gap between the trajectory and the object in the “Dark” plots in Figure 4. Less frequently, mice with intact whiskers inspected two adjacent faces of the cube by turning their head around the corner of the cube, close enough for the whiskers on one side of the snout to touch the cube, but again with little or no skin touch, a strategy we call “cheek brush” (Figure 5, middle left). These strategies were altered after whisker and microvibrissae removal. After trimming the macrovibrissae, snout and cheek brushes were no longer observed, and were replaced by “cheek presses,” in which the animal pressed its cheeks to the object while inspecting it from one or two sides (Figure 5, middle right). These changes are quantified in Figure 5 (lower panels) by pooling all reversal trials from 8 to 10 consecutive sessions under each of the first three sensory condition and from the two sessions without microvibrissae.

An especially prominent shift in strategy occurred immediately after microvibrissae depilation. This is illustrated in Figure 6, which plots the duration of object inspection for all side reversal trials in representative sessions from the four sensory conditions. As evident from this analysis, duration of object inspection fluctuated around five video frames (165 ms) under light, dark and trimmed whiskers conditions, but was substantially longer immediately after depilation of the microvibrissae (Figure 6, green lines). The initial trials in this condition consisted of extra-long cheek presses (up to 25 video frames, or >800 ms); the animal’s trajectory is illustrated in Figure 4 (“Trim and Nair: early”). In later trials within the same session the frequency of cheek presses was reduced, the mice reverted to inspecting the object with a brief snout touch (Figure 4, “Trim and Nair: late”) and duration of object inspection reverted back to baseline.

## Discussion

We report here that seven out of nine mice, trained in two cohorts on a custom-designed Y-maze, learned in about 20–35 daily sessions of 20 trials each to discriminate between two 3-D objects, using visual and tactile cues. Performance was only transiently degraded, if at all, when various sensory cues (light, whiskers, microvibrissae) were sequentially removed. These results are, to our knowledge, the first demonstration that mice can be successfully trained to discriminate between and recognize specific 3-D objects. While rats were previously trained on object discrimination tasks (Mumby et al., 1995; Vnek et al., 1995), it was not *a priori* clear that the same could be achieved in mice, because of potential inter-species differences in either perceptual abilities or learning prowess. Indeed, a lingering perception in the rodent behavior field is that mice are poor learners, compared to rats (Crawley, 1999). However, as already noted by others (Carandini and Churchland, 2013), rats may appear easier to train only because researchers have not yet discovered the appropriate techniques for training mice. In recent years the mouse has become the species of choice for studying the neural basis of behavior (Carandini and Churchland, 2013; Feldmeyer et al., 2013; Glickfeld et al., 2014). Newly developed genetic and viral tools allow investigators to record, image and manipulate activity in defined neuronal populations in the mouse brain with unprecedented spatial and temporal resolution (Alivisatos et al., 2013). Developing techniques for training mice in various perceptual and cognitive tasks is therefore highly desirable, and our results are a step in this direction. Specifically, the operant conditioning method we describe here opens up the study of the neural basis of object perception and recognition to these powerful techniques.

Formally, our behavioral task can be classified as a 3-D object discrimination task. In previous 3-D object discrimination studies, rats achieved criterion in about 50–300 trials, depending on study (Mumby et al., 1990; Vnek et al., 1995; Clark et al., 2001). While mice in our experiments required more trials to reach criterion, the rat studies were performed in full light and used objects which were highly distinct from each other. Our experiments were done under low light conditions and the task was (intentionally) challenging, given that the two objects (cube and tetrahedron) shared many overlapping features (height, color, texture, surface curvature). It is likely that learning would have proceeded faster with more distinct objects (Burke et al., 2011). Perhaps more importantly, our training arena differed from that used in the previous studies, in which rats had visual access to both objects simultaneously. With the possible exception of mouse B under red light conditions, mice in our experiments did not directly compare the two objects, and made their decisions by sampling the objects one at a time at close range, presumably using some combination of visual and tactile cues. Moreover, when by chance the mouse approached the correct object first, it immediately proceeded to collect the reward, without inspecting the other object. Thus, the animal’s decision did not rely on a direct comparison of the two objects, nor was it reached by retaining object information in working memory. Indeed, it is difficult to avoid the conclusion that the mice in our study reached their decisions by comparing the inspected object with a stored mental template of the object or a subset of its features; in other words, that the mice learned to *recognize* the objects. Thus, while formally testing object discrimination, our experiments likely engaged the neural networks for object recognition. Future studies could use methods previously employed in the rodent (Sakaguchi and Hayashi, 2012; Mayford, 2014; Brown and Banks, 2015; Tonegawa et al., 2015) to look for the hypothesized neuronal ensemble underlying the mental template of the object, i.e., the object engram.

It is worthwhile mentioning that many previous studies trained rats to discriminate between 2-D patterns or shapes of varying complexity. Such studies have a long history, dating back to Lashley (reviewed by Zoccolan, 2015). More recently, 2-D visual discrimination tasks were implemented on touch screens and applied to both mice and rats (Bussey et al., 2008; Horner et al., 2013). While this method allows automation of the training procedure and thereby greatly increases experimental throughput, these studies by their nature rely solely on visual cues, and thereby do not engage the full range of sensory modalities used by rodents (and primates) when exploring 3-D objects, nor are they likely to engage the brain regions in which multimodal object percepts are thought to be encoded (Amedi et al., 2005; Taylor et al., 2006; Lacey and Sathian, 2014; Burke and Barnes, 2015; Man et al., 2015; Jacklin et al., 2016).

While our study was designed as a proof-of-concept and our dataset to-date is still small (five animals in the pilot cohort and four in the main cohort), our observations offer insights into the perceptual and cognitive underpinnings of object identification in the mouse. One such insight is that, like other species including humans, individual mice vary considerably in their learning abilities (Matzel et al., 2003; Gallistel et al., 2004; Bathellier et al., 2013). Our fastest learner acquired the task after 30 daily sessions of 20 trials each, while the slowest learner still did not learn the task after nearly twice the number of sessions. Large inter-individual cognitive variability is found even within inbred mouse strains: in a study in which C57BL/6J mice were trained in vibrotactile discrimination, one animal reached criterion after one session while a second animal only after 31 sessions (Mayrhofer et al., 2013). A failure of one in four to five animals to achieve criterion is also typical in operant conditioning of mice, and is within the range of previous studies (e.g., Histed et al., 2012; Manita et al., 2015). Individual variability notwithstanding, the average learning curves of both cohorts are quite similar (Figure 2): for the first 15 sessions or so performance remained relatively flat, and thereafter average performance rose monotonously until reaching criterion. The flat part of the curve likely reflects the period before the animals realized that they need to pay attention to the objects, and were therefore performing at chance level, while the sloped part reflects the process of gradually learning which object is associated with the reward (see, for example, Figure 2 of Kerekes et al., 2017, for very similar learning curves of tactile discrimination training in rats).

Perhaps the most interesting insight from our study relates to the individualized strategies used by the animals to perform the object discrimination task, and the behavioral flexibility they displayed when various sensory cues were eliminated. Like rats (Huston and Aggleton, 1987), our mice used all sensory modalities available to them to explore the objects. Under orange-red light, which is only dimly visible to mice (Lyubarsky et al., 1999; Jacobs et al., 2004; Peirson et al., 2017), two of the animals relied on short-range tactile and (to some extent) visual cues, while the third animal relied on long-range visual cues, as judged by the observation that it nearly always approached the correct object first. When all visible light was turned off, performance of the first two animals dipped below criterion level but recovered within 5 and 20 sessions, respectively, suggesting that under red light these animals did rely to some extent on visual cues, but in total darkness gradually learned to use purely tactile cues. In contrast, performance of the third animal dipped below criterion for one session only; presumably it was able to switch almost immediately from purely visual to purely tactile cues. That under red light it was indeed using long-range visual (and not olfactory) cues was confirmed by the observation that in total darkness it switched to approaching either object first, at random. Of note, rats have previously been shown to switch between tactile and visual modalities in cross-modal object recognition studies (Winters and Reid, 2010; Hindley et al., 2014). Next, when a major source of tactile cues, the macrovibrissae, was eliminated, performance dipped transiently below criterion but again recovered within a few sessions. A similarly transient effect of whisker trimming on texture discrimination was previously reported in rats (Kerekes et al., 2017). Lastly, depilating the microvibrissae in two animals (in addition to trimming the macrovibrissae) did not degrade performance, but elicited a pronounced change in object inspection strategy, from short snout touches to prolonged cheek presses against one or two sides of the object. This change was, however, transient; in later trials within the same session, durations of object inspections were gradually shortened, eventually approaching durations with intact microvibrissae (typically <0.2 s). In future studies it would be of interest to test how well mice learn the task if from the onset they are trained in total darkness and/or deprived of vibrissae.

How did the mice tell the cube and tetrahedron apart in total darkness, when deprived of both macro- and micro-vibrissae? While we cannot totally rule out olfactory cues, it is unlikely that the objects differed in their olfactory signatures, as they were made from the same plastic material and printed with the same 3-D printer. In addition, objects were cleaned in 70% alcohol before and after each session, and during each session both objects were manipulated by the experimenter to an equal degree (and with gloved hands) when switching sides. Most likely, the mice were using the mechanoreceptors embedded in the whisker follicles or in the skin between and around the whisker follicles, receptors which presumably remained intact. Of the various mechanosensory receptor end organs, Merkel disks are necessary for slowly adapting light touch responses, and are thought to underlie the ability to recognize contours and edges of objects (Johnson, 2001; Nakatani et al., 2015; Severson et al., 2017). It would be interesting to determine whether mice in which functional Merkel cells were genetically ablated (Maricich et al., 2012) would still be able to discriminate between objects in the absence of macro- and micro-vibrissae. More generally, our development of a trained object discrimination paradigm for mice opens up the possibility of using the many genetically modified mouse strains available to examine the cellular and molecular basis of object perception.

Our home-made arena can be easily reproduced using inexpensive materials and standard machine shop tools. However, our system was not automated and required a human experimenter to be present during training and testing to retrieve the animal from the reward zone, to reshuffle the positions of the objects, to move or replace the food reward and to restart the next trial. In future studies, the training arena can be upgraded for semi- or fully-automated operation by adding return paths through which the mice would return spontaneously to the start point after each trial, and by incorporating sensor-triggered doors, object switchers and reward dispensers controlled by microelectronic devices (Sanders and Kepecs, 2014; Manita et al., 2015; Nashaat et al., 2016; Kerekes et al., 2017). Automating the system will allow training more animals simultaneously and running more trials per session. It will also allow the experimenter to monitor the training remotely, removing potential distractions and inadvertent cues associated with the presence of the experimenter in the room. Lastly, incorporation of high-resolution, high-speed digital cameras, with appropriate image-analysis algorithms designed for freely moving animals (Knutsen et al., 2005; Mitchinson et al., 2007; Voigts et al., 2008; Schroeder and Ritt, 2016), may allow tracking of whisker trajectories and a finer analysis of the strategies used by mice during tactile object exploration, recognition and discrimination.

## Data Availability

All the data reported in this manuscript are stored on the authors’ servers and are available upon request.

## Ethics Statement

This study was carried out in accordance with the guidelines of the United States Public Health Service.’ The protocol was approved by the West Virginia University Animal Care and Use Committee.

## Author Contributions

XH trained the main cohort and analyzed data. OU developed the methods. KC trained the pilot cohort and analyzed data. RH analyzed data. AA designed the study, analyzed data, and wrote the manuscript.

## Conflict of Interest Statement

The authors declare that the research was conducted in the absence of any commercial or financial relationships that could be construed as a potential conflict of interest.

## Footnotes

1 https://www.jax.org/jax-mice-and-services/strain-data-sheet-pages/body-weight-chart-000664

2 https://www.noldus.com/animal-behavior-research/products/ethovision-xt

## References

[B1] Aggleton J. P. Hunt P. R. Rawlins J. N. (1986). The effects of hippocampal lesions upon spatial and non-spatial tests of working memory. *Behav. Brain Res.* 19 133–146. 10.1016/0166-4328(86)90011-2 3964405

[B2] Akkerman S. Blokland A. Reneerkens O. van Goethem N. P. Bollen E. Gijselaers H. J. (2012). Object recognition testing: methodological considerations on exploration and discrimination measures. *Behav. Brain Res.* 232 335–347. 10.1016/j.bbr.2012.03.022 22490364

[B3] Alivisatos A. P. Andrews A. M. Boyden E. S. Chun M. Church G. M. Deisseroth K. (2013). Nanotools for neuroscience and brain activity mapping. *ACS Nano* 7 1850–1866. 10.1021/nn4012847 23514423 PMC3665747

[B4] Allen W. E. Kauvar I. V. Chen M. Z. Richman E. B. Yang S. J. Chan K. (2017). Global representations of goal-directed behavior in distinct cell types of mouse neocortex. *Neuron* 94 891–907.e6. 10.1016/j.neuron.2017.04.017 28521139 PMC5723385

[B5] Amedi A. von Kriegstein K. van Atteveldt N. M. Beauchamp M. S. Naumer M. J. (2005). Functional imaging of human crossmodal identification and object recognition. *Exp. Brain Res.* 166 559–571. 10.1007/s00221-005-2396-5 16028028

[B6] Anjum F. Turni H. Mulder P. G. van der Burg J. Brecht M. (2006). Tactile guidance of prey capture in Etruscan shrews. *Proc. Natl. Acad. Sci. U.S.A.* 103 16544–16549. 10.1073/pnas.0605573103 17060642 PMC1621049

[B7] Antunes M. Biala G. (2012). The novel object recognition memory: neurobiology, test procedure, and its modifications. *Cogn. Process.* 13 93–110. 10.1007/s10339-011-0430-z 22160349 PMC3332351

[B8] Bathellier B. Tee S. P. Hrovat C. Rumpel S. (2013). A multiplicative reinforcement learning model capturing learning dynamics and interindividual variability in mice. *Proc. Natl. Acad. Sci. U.S.A.* 110 19950–19955. 10.1073/pnas.1312125110 24255115 PMC3856837

[B9] Blaser R. Heyser C. (2015). Spontaneous object recognition: a promising approach to the comparative study of memory. *Front. Behav. Neurosci.* 9:183. 10.3389/fnbeh.2015.00183 26217207 PMC4498097

[B10] Brecht M. Preilowski B. Merzenich M. M. (1997). Functional architecture of the mystacial vibrissae. *Behav. Brain Res.* 84 81–97. 10.1016/S0166-4328(97)83328-1 9079775

[B11] Broadbent N. J. Gaskin S. Squire L. R. Clark R. E. (2010). Object recognition memory and the rodent hippocampus. *Learn. Mem.* 17 5–11. 10.1101/lm.1650110 20028732 PMC2807177

[B12] Brown M. W. Banks P. J. (2015). In search of a recognition memory engram. *Neurosci. Biobehav. Rev.* 50 12–28. 10.1016/j.neubiorev.2014.09.016 25280908 PMC4382520

[B13] Brown M. W. Barker G. R. Aggleton J. P. Warburton E. C. (2012). What pharmacological interventions indicate concerning the role of the perirhinal cortex in recognition memory. *Neuropsychologia* 50 3122–3140. 10.1016/j.neuropsychologia.2012.07.034 22841990 PMC3500694

[B14] Bulthoff H. H. Edelman S. (1992). Psychophysical support for a two-dimensional view interpolation theory of object recognition. *Proc. Natl. Acad. Sci. U.S.A.* 89 60–64. 10.1073/pnas.89.1.60 1729718 PMC48175

[B15] Burke S. N. Barnes C. A. (2015). The neural representation of 3-dimensional objects in rodent memory circuits. *Behav. Brain Res.* 285 60–66. 10.1016/j.bbr.2014.09.001 25205370 PMC4362856

[B16] Burke S. N. Wallace J. L. Hartzell A. L. Nematollahi S. Plange K. Barnes C. A. (2011). Age-associated deficits in pattern separation functions of the perirhinal cortex: a cross-species consensus. *Behav. Neurosci.* 125 836–847. 10.1037/a0026238 22122147 PMC3255096

[B17] Bussey T. J. Padain T. L. Skillings E. A. Winters B. D. Morton A. J. Saksida L. M. (2008). The touchscreen cognitive testing method for rodents: how to get the best out of your rat. *Learn. Mem.* 15 516–523. 10.1101/lm.987808 18612068 PMC2505319

[B18] Carandini M. Churchland A. K. (2013). Probing perceptual decisions in rodents. *Nat. Neurosci.* 16 824–831. 10.1038/nn.3410 23799475 PMC4105200

[B19] Catania K. C. (2012). Tactile sensing in specialized predators - from behavior to the brain. *Curr. Opin. Neurobiol.* 22 251–258. 10.1016/j.conb.2011.11.014 22209039

[B20] Clark R. E. West A. N. Zola S. M. Squire L. R. (2001). Rats with lesions of the hippocampus are impaired on the delayed nonmatching-to-sample task. *Hippocampus* 11 176–186. 10.1002/hipo.1035 11345124

[B21] Cohen S. J. Stackman R. W. Jr. (2015). Assessing rodent hippocampal involvement in the novel object recognition task. A review. *Behav. Brain Res.* 285 105–117. 10.1016/j.bbr.2014.08.002 25169255 PMC7008635

[B22] Cole E. Simundic A. Mossa F. P. Mumby D. G. (2018). Assessing object-recognition memory in rats: pitfalls of the existent tasks and the advantages of a new test. *Learn. Behav.* 10.3758/s13420-018-0347-9 [Epub ahead of print]. 30132280

[B23] Crawley J. N. (1999). Behavioral phenotyping of transgenic and knockout mice: experimental design and evaluation of general health, sensory functions, motor abilities, and specific behavioral tests. *Brain Res.* 835 18–26. 10.1016/S0006-8993(98)01258-X 10448192

[B24] Dere E. Huston J. P. De Souza Silva M. A. (2007). The pharmacology, neuroanatomy and neurogenetics of one-trial object recognition in rodents. *Neurosci. Biobehav. Rev.* 31 673–704. 10.1016/j.neubiorev.2007.01.005 17368764

[B25] Diamond M. E. von Heimendahl M. Knutsen P. M. Kleinfeld D. Ahissar E. (2008). ‘Where’ and ‘what’ in the whisker sensorimotor system. *Nat. Rev. Neurosci.* 9 601–612. 10.1038/nrn2411 18641667

[B26] DiCarlo J. J. Zoccolan D. Rust N. C. (2012). How does the brain solve visual object recognition? *Neuron* 73 415–434. 10.1016/j.neuron.2012.01.010 22325196 PMC3306444

[B27] Ennaceur A. Cavoy A. Costa J. C. Delacour J. (1989). A new one-trial test for neurobiological studies of memory in rats. II: effects of piracetam and pramiracetam. *Behav. Brain Res.* 33 197–207. 10.1016/S0166-4328(89)80051-8 2765166

[B28] Ennaceur A. Delacour J. (1988). A new one-trial test for neurobiological studies of memory in rats. 1: behavioral data. *Behav. Brain Res.* 31 47–59. 10.1016/0166-4328(88)90157-X 3228475

[B29] Ennaceur A. Meliani K. (1992). A new one-trial test for neurobiological studies of memory in rats. III. Spatial vs. non-spatial working memory. *Behav. Brain Res.* 51 83–92. 10.1016/S0166-4328(05)80315-8 1482548

[B30] Feldmeyer D. Brecht M. Helmchen F. Petersen C. C. Poulet J. F. Staiger J. F. (2013). Barrel cortex function. *Prog. Neurobiol.* 103 3–27. 10.1016/j.pneurobio.2012.11.002 23195880

[B31] Fellows B. J. (1967). Chance stimulus sequences for discrimination tasks. *Psychol. Bull.* 67 87–92. 10.1037/h0024098 6045339

[B32] Fu Y. Rusznak Z. Herculano-Houzel S. Watson C. Paxinos G. (2013). Cellular composition characterizing postnatal development and maturation of the mouse brain and spinal cord. *Brain Struct. Funct.* 218 1337–1354. 10.1007/s00429-012-0462-x 23052551

[B33] Fuster J. M. (2000). Executive frontal functions. *Exp. Brain Res.* 133 66–70. 10.1007/s002210000401 10933211

[B34] Gallistel C. R. Fairhurst S. Balsam P. (2004). The learning curve: implications of a quantitative analysis. *Proc. Natl. Acad. Sci. U.S.A.* 101 13124–13131. 10.1073/pnas.0404965101 15331782 PMC516535

[B35] Gaskin S. Tardif M. Cole E. Piterkin P. Kayello L. Mumby D. G. (2010). Object familiarization and novel-object preference in rats. *Behav. Processes* 83 61–71. 10.1016/j.beproc.2009.10.003 19874876

[B36] Gellerman L. W. (1933). Chance orders of alternating stimuli in visual discrimination experiments. *J. Genet. Psychol.* 42 206–208. 10.1080/08856559.1933.10534237

[B37] Gervais N. J. Jacob S. Brake W. G. Mumby D. G. (2013). Systemic and intra-rhinal-cortical 17-beta estradiol administration modulate object-recognition memory in ovariectomized female rats. *Horm. Behav.* 64 642–652. 10.1016/j.yhbeh.2013.08.010 24012943

[B38] Glickfeld L. L. Reid R. C. Andermann M. L. (2014). A mouse model of higher visual cortical function. *Curr. Opin. Neurobiol.* 24 28–33. 10.1016/j.conb.2013.08.009 24492075 PMC4398969

[B39] Goldman-Rakic P. S. (1996). Regional and cellular fractionation of working memory. *Proc. Natl. Acad. Sci. U.S.A.* 93 13473–13480. 10.1073/pnas.93.24.13473 8942959 PMC33633

[B40] Guo Z. V. Li N. Huber D. Ophir E. Gutnisky D. Ting J. T. (2014). Flow of cortical activity underlying a tactile decision in mice. *Neuron* 81 179–194. 10.1016/j.neuron.2013.10.020 24361077 PMC3984938

[B41] Gurtubay-Antolin A. Rodriguez-Herreros B. Rodriguez-Fornells A. (2015). The speed of object recognition from a haptic glance: event-related potential evidence. *J. Neurophysiol.* 113 3069–3075. 10.1152/jn.00836.2014 25744887 PMC4455565

[B42] Harvey C. D. Coen P. Tank D. W. (2012). Choice-specific sequences in parietal cortex during a virtual-navigation decision task. *Nature* 484 62–68. 10.1038/nature10918 22419153 PMC3321074

[B43] Hindley E. L. Nelson A. J. Aggleton J. P. Vann S. D. (2014). Dysgranular retrosplenial cortex lesions in rats disrupt cross-modal object recognition. *Learn. Mem.* 21 171–179. 10.1101/lm.032516.113 24554671 PMC3929849

[B44] Hirabayashi T. Miyashita Y. (2014). Computational principles of microcircuits for visual object processing in the macaque temporal cortex. *Trends Neurosci.* 37 178–187. 10.1016/j.tins.2014.01.002 24491832

[B45] Histed M. H. Carvalho L. A. Maunsell J. H. (2012). Psychophysical measurement of contrast sensitivity in the behaving mouse. *J. Neurophysiol.* 107 758–765. 10.1152/jn.00609.2011 22049334 PMC3289478

[B46] Hoffman K. L. Logothetis N. K. (2009). Cortical mechanisms of sensory learning and object recognition. *Philos. Trans. R. Soc. Lond. Ser. B Biol. Sci.* 364 321–329. 10.1098/rstb.2008.0271 18977728 PMC2674481

[B47] Horner A. E. Heath C. J. Hvoslef-Eide M. Kent B. A. Kim C. H. Nilsson S. R. (2013). The touchscreen operant platform for testing learning and memory in rats and mice. *Nat. Protoc.* 8 1961–1984. 10.1038/nprot.2013.122 24051959 PMC3914026

[B48] Huberman A. D. Niell C. M. (2011). What can mice tell us about how vision works? *Trends Neurosci.* 34 464–473. 10.1016/j.tins.2011.07.002 21840069 PMC3371366

[B49] Hung C. P. Kreiman G. Poggio T. DiCarlo J. J. (2005). Fast readout of object identity from macaque inferior temporal cortex. *Science* 310 863–866. 10.1126/science.1117593 16272124

[B50] Huston A. E. Aggleton J. P. (1987). The effects of cholinergic drugs upon recognition memory in rats. *Q. J. Exp. Psychol. B Compar. Physiol. Psychol.* 39 297–314. 2829289

[B51] Jacklin D. L. Cloke J. M. Potvin A. Garrett I. Winters B. D. (2016). The dynamic multisensory engram: neural circuitry underlying crossmodal object recognition in rats changes with the nature of object experience. *J. Neurosci.* 36 1273–1289. 10.1523/JNEUROSCI.3043-15.2016 26818515 PMC6604816

[B52] Jackson-Smith P. Kesner R. P. Chiba A. A. (1993). Continuous recognition of spatial and nonspatial stimuli in hippocampal-lesioned rats. *Behav. Neural Biol.* 59 107–119. 10.1016/0163-1047(93)90821-X 8476378

[B53] Jacobs G. H. Williams G. A. Fenwick J. A. (2004). Influence of cone pigment coexpression on spectral sensitivity and color vision in the mouse. *Vis. Res.* 44 1615–1622. 10.1016/j.visres.2004.01.016 15135998

[B54] Johnson K. O. (2001). The roles and functions of cutaneous mechanoreceptors. *Curr. Opin. Neurobiol.* 11 455–461. 10.1016/S0959-4388(00)00234-8 11502392

[B55] Kerekes P. Daret A. Shulz D. E. Ego-Stengel V. (2017). Bilateral discrimination of tactile patterns without whisking in freely running rats. *J. Neurosci.* 37 7567–7579. 10.1523/JNEUROSCI.0528-17.2017 28663200 PMC6596651

[B56] Knutsen P. M. Derdikman D. Ahissar E. (2005). Tracking whisker and head movements in unrestrained behaving rodents. *J. Neurophysiol.* 93 2294–2301. 10.1152/jn.00718.2004 15563552

[B57] Kuruppath P. Gugig E. Azouz R. (2014). Microvibrissae-based texture discrimination. *J. Neurosci.* 34 5115–5120. 10.1523/JNEUROSCI.4217-13.2014 24719091 PMC6608996

[B58] Lacey S. Sathian K. (2014). Visuo-haptic multisensory object recognition, categorization, and representation. *Front. Psychol.* 5:730. 10.3389/fpsyg.2014.00730 25101014 PMC4102085

[B59] Levine M. (1963). Mediating processes in humans at the outset of discrimination learning. *Psychol. Rev.* 70 254–276. 10.1037/h0045543 13930155

[B60] Liu H. Agam Y. Madsen J. R. Kreiman G. (2009). Timing, timing, timing: fast decoding of object information from intracranial field potentials in human visual cortex. *Neuron* 62 281–290. 10.1016/j.neuron.2009.02.025 19409272 PMC2921507

[B61] Logothetis N. K. Sheinberg D. L. (1996). Visual object recognition. *Annu. Rev. Neurosci.* 19 577–621. 10.1146/annurev.ne.19.030196.003045 8833455

[B62] Lyubarsky A. L. Falsini B. Pennesi M. E. Valentini P. Pugh E. N. Jr. (1999). UV- and midwave-sensitive cone-driven retinal responses of the mouse: a possible phenotype for coexpression of cone photopigments. *J. Neurosci.* 19 442–455. 10.1523/JNEUROSCI.19-01-00442.1999 9870972 PMC6782392

[B63] Man K. Damasio A. Meyer K. Kaplan J. T. (2015). Convergent and invariant object representations for sight, sound, and touch. *Hum. Brain Mapp.* 36 3629–3640. 10.1002/hbm.22867 26047030 PMC6869094

[B64] Manita S. Suzuki T. Homma C. Matsumoto T. Odagawa M. Yamada K. (2015). A top-down cortical circuit for accurate sensory perception. *Neuron* 86 1304–1316. 10.1016/j.neuron.2015.05.006 26004915

[B65] Maricich S. M. Morrison K. M. Mathes E. L. Brewer B. M. (2012). Rodents rely on Merkel cells for texture discrimination tasks. *J. Neurosci.* 32 3296–3300. 10.1523/JNEUROSCI.5307-11.2012 22399751 PMC3306053

[B66] Matzel L. D. Han Y. R. Grossman H. Karnik M. S. Patel D. Scott N. (2003). Individual differences in the expression of a “general” learning ability in mice. *J. Neurosci.* 23 6423–6433. 10.1523/JNEUROSCI.23-16-06423.2003 12878682 PMC6740645

[B67] Mayford M. (2014). The search for a hippocampal engram. *Philos. Trans. R. Soc. Lond. Ser. B Biol. Sci.* 369:20130161. 10.1098/rstb.2013.0161 24298162 PMC3843892

[B68] Mayrhofer J. M. Skreb V. von der Behrens W. Musall S. Weber B. Haiss F. (2013). Novel two-alternative forced choice paradigm for bilateral vibrotactile whisker frequency discrimination in head-fixed mice and rats. *J. Neurophysiol.* 109 273–284. 10.1152/jn.00488.2012 23054598

[B69] Mishkin M. Delacour J. (1975). An analysis of short-term visual memory in the monkey. *J. Exp. Psychol. Anim. Behav. Process.* 1 326–334. 10.1037/0097-7403.1.4.326 811754

[B70] Mishkin M. Prockop E. S. Rosvold H. E. (1962). One-trial object discrimination learning in monkeys with frontal lesions. *J. Compar. Physiol. Psychol.* 55 178–181. 10.1037/h0046525 14474552

[B71] Mitchinson B. Martin C. J. Grant R. A. Prescott T. J. (2007). Feedback control in active sensing: rat exploratory whisking is modulated by environmental contact. *Proc. Biol. Sci.* 274 1035–1041. 10.1098/rspb.2006.0347 17331893 PMC2124479

[B72] Mumby D. G. Astur R. S. Weisend M. P. Sutherland R. J. (1999). Retrograde amnesia and selective damage to the hippocampal formation: memory for places and object discriminations. *Behav. Brain Res.* 106 97–107. 10.1016/S0166-4328(99)00097-2 10595425

[B73] Mumby D. G. Glenn M. J. Nesbitt C. Kyriazis D. A. (2002). Dissociation in retrograde memory for object discriminations and object recognition in rats with perirhinal cortex damage. *Behav. Brain Res.* 132 215–226. 10.1016/S0166-4328(01)00444-2 11997151

[B74] Mumby D. G. Pinel J. P. Kornecook T. J. Shen M. J. Redila V. A. (1995). Memory deficits following lesions of hippocampus or amygdala in rat: assessment by an object-memory test battery. *Psychobiology* 23 26–36.

[B75] Mumby D. G. Pinel J. P. Wood E. R. (1990). Nonrecurring-items delayed nonmatching-to-sample in rats: a new paradigm for testing nonspatial working memory. *Psychobiology* 18 321–326.

[B76] Nakatani M. Maksimovic S. Baba Y. Lumpkin E. A. (2015). Mechanotransduction in epidermal Merkel cells. *Pflugers Arch.* 467 101–108. 10.1007/s00424-014-1569-0 25053537 PMC4282617

[B77] Nashaat M. A. Oraby H. Sachdev R. N. Winter Y. Larkum M. E. (2016). Air-Track: a real-world floating environment for active sensing in head-fixed mice. *J. Neurophysiol.* 116 1542–1553. 10.1152/jn.00088.2016 27486102 PMC5144720

[B78] O’Connor D. H. Huber D. Svoboda K. (2009). Reverse engineering the mouse brain. *Nature* 461 923–929. 10.1038/nature08539 19829372

[B79] Op de Beeck H. P. Baker C. I. (2010). The neural basis of visual object learning. *Trends Cogn. Sci.* 14 22–30. 10.1016/j.tics.2009.11.002 19945336 PMC2818494

[B80] Palmeri T. J. Gauthier I. (2004). Visual object understanding. *Nat. Rev. Neurosci.* 5 291–303. 10.1038/nrn1364 15034554

[B81] Peirson S. N. Brown L. A. Pothecary C. A. Benson L. A. Fisk A. S. (2017). Light and the laboratory mouse. *J. Neurosci. Methods* 300 26–36. 10.1016/j.jneumeth.2017.04.007 28414048 PMC5909038

[B82] Petersen C. C. Crochet S. (2013). Synaptic computation and sensory processing in neocortical layer 2/3. *Neuron* 78 28–48. 10.1016/j.neuron.2013.03.020 23583106

[B83] Riesenhuber M. Poggio T. (2002). Neural mechanisms of object recognition. *Curr. Opin. Neurobiol.* 12 162–168. 10.1016/S0959-4388(02)00304-5 12015232

[B84] Romo R. Salinas E. (2003). Flutter discrimination: neural codes, perception, memory and decision making. *Nat. Rev. Neurosci.* 4 203–218. 10.1038/nrn1058 12612633

[B85] Roohbakhsh A. Shamsizadeh A. Arababadi M. K. Ayoobi F. Fatemi I. Allahtavakoli M. (2016). Tactile learning in rodents: neurobiology and neuropharmacology. *Life Sci.* 147 1–8. 10.1016/j.lfs.2016.01.031 26800784

[B86] Rothblat L. A. Hayes L. L. (1987). Short-term object recognition memory in the rat: nonmatching with trial-unique junk stimuli. *Behav. Neurosci.* 101 587–590. 10.1037/0735-7044.101.4.587 3651236

[B87] Sakaguchi M. Hayashi Y. (2012). Catching the engram: strategies to examine the memory trace. *Mol. Brain* 5:32. 10.1186/1756-6606-5-32 22999350 PMC3462696

[B88] Sanders J. I. Kepecs A. (2014). A low-cost programmable pulse generator for physiology and behavior. *Front. Neuroeng.* 7:43. 10.3389/fneng.2014.00043 25566051 PMC4263096

[B89] Schroeder J. B. Ritt J. T. (2016). Selection of head and whisker coordination strategies during goal-oriented active touch. *J. Neurophysiol.* 115 1797–1809. 10.1152/jn.00465.2015 26792880 PMC4869481

[B90] Serre T. Kreiman G. Kouh M. Cadieu C. Knoblich U. Poggio T. (2007). A quantitative theory of immediate visual recognition. *Prog. Brain Res.* 165 33–56. 10.1016/S0079-6123(06)65004-8 17925239

[B91] Severson K. S. Xu D. Van de Loo M. Bai L. Ginty D. D. O’Connor D. H. (2017). Active touch and self-motion encoding by merkel cell-associated afferents. *Neuron* 94 666–676e669. 10.1016/j.neuron.2017.03.045 28434802 PMC5528144

[B92] Shadlen M. N. Kiani R. (2013). Decision making as a window on cognition. *Neuron* 80 791–806. 10.1016/j.neuron.2013.10.047 24183028 PMC3852636

[B93] Soto F. A. Wasserman E. A. (2014). Mechanisms of object recognition: what we have learned from pigeons. *Front. Neural Circ.* 8:122. 10.3389/fncir.2014.00122 25352784 PMC4195317

[B94] Tarr M. J. Bulthoff H. H. (1998). Image-based object recognition in man, monkey and machine. *Cognition* 67 1–20. 10.1016/S0010-0277(98)00026-2 9735534

[B95] Taylor K. I. Moss H. E. Stamatakis E. A. Tyler L. K. (2006). Binding crossmodal object features in perirhinal cortex. *Proc. Natl. Acad. Sci. U.S.A.* 103 8239–8244. 10.1073/pnas.0509704103 16702554 PMC1461402

[B96] Thorpe S. Fize D. Marlot C. (1996). Speed of processing in the human visual system. *Nature* 381 520–522. 10.1038/381520a0 8632824

[B97] Tonegawa S. Liu X. Ramirez S. Redondo R. (2015). Memory engram cells have come of age. *Neuron* 87 918–931. 10.1016/j.neuron.2015.08.002 26335640

[B98] Ungerleider L. G. Bell A. H. (2011). Uncovering the visual “alphabet”: advances in our understanding of object perception. *Vis. Res.* 51 782–799. 10.1016/j.visres.2010.10.002 20971130 PMC3208055

[B99] Vnek N. Gleason T. C. Kromer L. F. Rothblat L. A. (1995). Entorhinal-hippocampal connections and object memory in the rat: acquisition versus retention. *J. Neurosci.* 15 3193–3199. 10.1523/JNEUROSCI.15-04-03193.1995 7722656 PMC6577796

[B100] Voigts J. Sakmann B. Celikel T. (2008). Unsupervised whisker tracking in unrestrained behaving animals. *J. Neurophysiol.* 100 504–515. 10.1152/jn.00012.2008 18463190

[B101] Warburton E. C. Brown M. W. (2015). Neural circuitry for rat recognition memory. *Behav. Brain Res.* 285 131–139. 10.1016/j.bbr.2014.09.050 25315129 PMC4383363

[B102] Winters B. D. Reid J. M. (2010). A distributed cortical representation underlies crossmodal object recognition in rats. *J. Neurosci.* 30 6253–6261. 10.1523/JNEUROSCI.6073-09.2010 20445051 PMC6632708

[B103] Winters B. D. Saksida L. M. Bussey T. J. (2008). Object recognition memory: neurobiological mechanisms of encoding, consolidation and retrieval. *Neurosci. Biobehav. Rev.* 32 1055–1070. 10.1016/j.neubiorev.2008.04.004 18499253

[B104] Zeng H. Madisen L. (2012). Mouse transgenic approaches in optogenetics. *Prog. Brain Res.* 196 193–213. 10.1016/B978-0-444-59426-6.00010-0 22341327 PMC3433654

[B105] Zoccolan D. (2015). Invariant visual object recognition and shape processing in rats. *Behav. Brain Res.* 285 10–33. 10.1016/j.bbr.2014.12.053 25561421 PMC4383365

[B106] Zola-Morgan S. Squire L. R. Amaral D. G. (1989). Lesions of the hippocampal formation but not lesions of the fornix or the mammillary nuclei produce long-lasting memory impairment in monkeys. *J. Neurosci.* 9 898–913. 10.1523/JNEUROSCI.09-03-00898.1989 2494309 PMC6569954

